# *Selaginella moellendorffii* telomeres: conserved and unique features in an ancient land plant lineage

**DOI:** 10.3389/fpls.2012.00161

**Published:** 2012-07-19

**Authors:** Eugene V. Shakirov, Dorothy E. Shippen

**Affiliations:** ^1^Department of Biology, Texas A&M University, College Station, TX, USA; ^2^Department of Biochemistry and Biophysics, Texas A&M University, College Station, TX, USA

**Keywords:** telomere, *Selaginella*, POT1, TRFL1, CST complex

## Abstract

Telomeres, the essential terminal regions of linear eukaryotic chromosomes, consist of G-rich DNA repeats bound by a plethora of associated proteins. While the general pathways of telomere maintenance are evolutionarily conserved, individual telomere complex components show remarkable variation between eukaryotic lineages and even within closely related species. The recent genome sequencing of the lycophyte *Selaginella moellendorffii* and the availability of an ever-increasing number of flowering plant genomes provides a unique opportunity to evaluate the molecular and functional evolution of telomere components from the early evolving non-seed plants to the more developmentally advanced angiosperms. Here we analyzed telomere sequence in *S. moellendorffii* and found it to consist of TTTAGGG repeats, typical of most plants. Telomere tracts in *S. moellendorffii* range from 1 to 5.5 kb, closely resembling *Arabidopsis thaliana*. We identified several *S. moellendorffii* genes encoding sequence homologs of proteins involved in telomere maintenance in other organisms, including CST complex components and the telomere-binding proteins, POT1 and the TRFL family. Notable sequence similarities and differences were uncovered among the telomere-related genes in some of the plant lineages. Taken together, the data indicate that comparative analysis of the telomere complex in early diverging land plants such as *S. moellendorffii* and green algae will yield important insights into the evolution of telomeres and their protein constituents.

## INTRODUCTION

The ends of linear eukaryotic chromosomes terminate with a long stretch of simple tandem repeats of GT-rich telomeric DNA. These sequences, together with specific DNA binding proteins, comprise the telomeres. Telomeres are important for maintaining genome integrity, distinguishing natural DNA ends from double-strand (ds) breaks, and preventing illegitimate DNA repair. Proper maintenance of telomeric DNA length and structure is essential for normal cell viability (Zellinger and Riha, 2007).

Eukaryotic organisms across different taxa display remarkable variation in the length of telomere tracts. Unicellular ciliates and budding yeast typically harbor short telomeres that range from several dozen to several hundred base pairs, while humans and mice have telomeres in the range of 5–15 kb and 10–60 kb, respectively (Hug and Lingner, 2006). Plants also display dramatic variations in telomere length, with tracts spanning 2–5 kb in *Arabidopsis thaliana* to >150 kb in tobacco (Richards and Ausubel, 1988; Fajkus et al., 1995; Shakirov and Shippen, 2004). In addition, telomere length varies not only from species to species, but even within different populations of the same species. Telomere length in different *A. thaliana* ecotypes (natural populations) varies as much as twofold (Shakirov and Shippen, 2004; Maillet et al., 2006), while some *Zea mays* recombinant inbred lines show up to 25-fold differences in telomere length (Burr et al., 1992).

Telomere binding proteins play essential roles in regulating telomere length by modulating telomerase access to chromosome ends. Numerous other proteins influence telomere length, including DNA damage response factors and DNA-modifying enzymes (Martinez and Blasco, 2011). Notably, a deletion screen of all non-essential genes in budding yeast identified ~200 candidates whose absence resulted in deregulated telomeres (Askree et al., 2004). While most of these genes likely affect telomere homeostasis indirectly, these genetic data underscore the dynamic and complex nature of telomere length regulation.

Plants and animals diverged over 1.5 bya (Yoon et al., 2004) and yet many aspects of telomere biology are conserved. For example, the most common telomere repeat sequence in plants is TTTAGGG, just one nucleotide longer than the 6-base sequence TTAGGG found in vertebrates (McKnight and Shippen, 2004). Many sequence and functional homologs of telomere-related genes in vertebrates and yeast have been identified in plants (Fitzgerald et al., 1999; Riha et al., 2002; Karamysheva et al., 2004; Shakirov et al., 2005; Song et al., 2008; Surovtseva et al., 2009). Indeed plants provide a unique opportunity to examine evolution of telomere composition, structure, and function due to the well-established evolutionary relationships within the plant kingdom. Here we exploit the recently sequenced genome of the lycophyte *Selaginella moellendorffii* (Banks et al., 2011) to characterize telomeric DNA and to identify genes with putative roles in telomere biology. Our analysis indicates that *S. moellendorffii* harbors short telomere tracts consisting of canonical TTTAGGG repeats. Furthermore, we find a full complement of the telomere-associated genes that have previously been described in other plants. Comparative studies of *S. moellendorffi* with other early diverging plants may be useful for studying the evolution of telomere proteins in plants.

## RESULTS AND DISCUSSION

### *SELAGINELLA MOELLENDORFFII* TELOMERES

**FIGURE 1:**
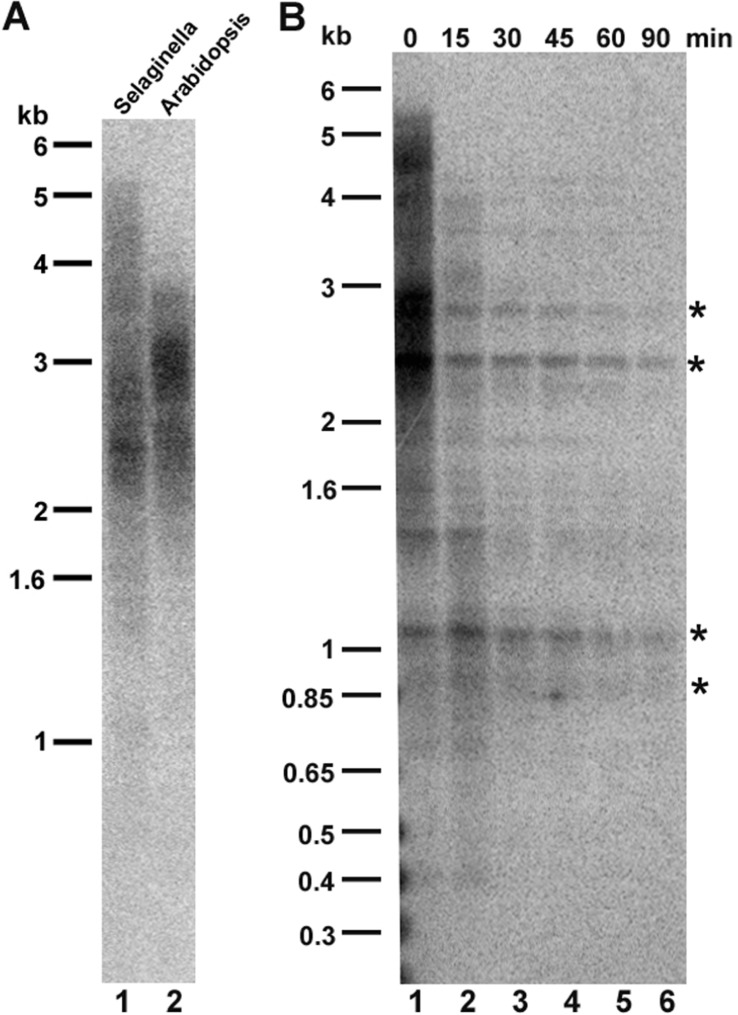
** Telomere length analysis in *Selaginella moellendorffii*.** **(A)** Comparative terminal restriction fragment (TRF) analysis of *S. moellendorffii* (lane 1) and *A. thaliana* (lane 2) telomeres. Molecular weight markers are shown on the left. **(B)** *Bal*31 digestion of *S. moellendorffii* telomeric DNA. Lane 1, *Tru*1I digestion of genomic DNA without prior *Bal*treatment (0 min). Lanes 2–6, *Tru*1I digestion of genomic DNA with *Bal*31 tr31eatment for 15, 30, 45, 60, and 90 min, respectively. Asterisks indicate cross-hybridizing interstitial telomeric DNA bands, which are not sensitive to *Bal*31 digestion for up to 90 min.

Sequence analysis of terminal chromosomal scaffolds indicates that *S. moellendorffii* telomeres, like those of most other plants, are composed of tandem arrays of (TTTAGGG)**_n_ repeats (Banks et al., 2011). To gauge the size of *S. moellendorffii* telomere tracts, we performed terminal restriction fragment (TRF) analysis using *Tru*11. The blot was hybridized with probe corresponding to four repeats of TTTAGGG. As shown in **Figure 1A**, *S. moellendorffii* telomere tracts migrated as a smear ranging from 1.5 to 5.5 kb, closely resembling telomere profile in many *A. thaliana* accessions (Richards and Ausubel, 1988; Shakirov and Shippen, 2004).

We verified that sequences detected by TRF analysis correspond to chromosome ends using the non-specific *Bal*31 exonuclease. *Bal*31 preferentially degrades DNA ends versus more internal genomic regions. DNA was pre-incubated with *Bal*31 prior to digestion with *Tru*1I, and a Southern blot was performed. After 15 min of *Bal*31 digestion, the hybridization products migrated faster on the gel and showed reduced intensity (**Figure 1B**, lane 2). With continued *Bal*31 incubation, the telomeric signal disappeared completely (**Figure 1B**, lanes 3–6). In contrast, several cross-hybridizing bands, corresponding to interstitial telomeric DNA were insensitive to *Bal*31 digestion for up to 90 min, supporting the conclusion that the *Bal*31-sensitive hybridization signal corresponds to terminal telomeric DNA. Thus, *S. moellendorffii* telomeres are comprised of 1.5–5.5 kb tracts of TTTAGG repeats.

### TELOMERE-RELATED GENES IN *S. MOELLENDORFFII*

#### POT1 proteins

Single-strand (ss) telomere-binding proteins represent a key component of the telomere cap. Such proteins control telomerase access to the telomere and ensure chromosome end protection (de Lange, 2009). Overall, ss telomere binding proteins share limited sequence similarity, but they all bear signature N-terminal oligonucleotide/oligosacchaaride folds (OB-folds). One key ss telomere binding protein is Protection of telomeres (POT1; Baumann and Cech, 2001). In the moss *Physcomitrella patens*, a single-copy *POT*1 gene encodes a typical DNA binding protein that efficiently binds ss telomeric substrates *in vitro* (Shakirov et al., 2010). Furthermore, similar to its mammalian and fission yeast counterparts, *P. patens* POT1 is involved in telomere end protection (capping). While *PpPOT*1-deficient moss can survive long-term in culture, the mutant strain is sterile and shows end-to-end chromosome fusions, indicating that the overall telomere protective function of POT1 is conserved bet-ween early diverging land plants and other eukaryotes (Shakirov et al., 2010).

Despite conservation of POT1 function in the earliest land plant lineages, several lines of biochemical and genetic evidence indicate that the functions of POT1 in vascular plants (starting with *S. moellendorffii*) may have changed substantially. First, biochemical analysis of POT1 proteins from 13 plants representing major evolutionary branches of plants has indicated that the ability to bind telomeric DNA has been lost for most plant POT1 proteins, including POT1 from *S. moellendorffii* (Shakirov et al., 2009a,b). In fact, besides the *P. patens* POT1 protein and its ortholog from the green alga *Ostreococcus lucimarinus*, only two other POT1 proteins (from *Asparagus officinalis* and *Z. mays*) out of a total of 16 surveyed have retained the capacity to bind telomeric DNA *in vitro* (Shakirov et al., 2009b). However, both *A. officinalis* and *Z. mays* are unusual plants with respect to telomere biology. *A. officinalis* possesses unconventional telomere repeats TTAGGG instead of the canonical TTTAGGG, while *Z. mays* belongs to the only plant family surveyed other than Brassicaceae that harbors duplicated *POT*1 genes (Shakirov et al., 2009a,b). Thus, the ability of POT1 proteins from *A. officinalis* and *Z. mays* to bind telomeric DNA may have been conserved due to unusual changes in organismal telomere biology (*A. officinalis*) or protein sub-functionalization (*Z. mays*). Alternatively, the ability of *A. officinalis* and *Z. mays* POT1 proteins to bind telomeric DNA may have evolved independently through parallel evolution.

The second line of evidence supporting unusually fast evolution of POT1 functions in vascular plants comes from the studies of *A. thaliana*. Unlike the situation in humans and most other organisms, *A. thaliana* and other members of the Brassicaceae family possess two full-length POT1 proteins (Shakirov et al., 2005, 2009b). Genetic and biochemical studies indicate that AtPOT1a is a positive regulator of telomere length working in the context of telomerase holoenzyme (Surovtseva et al., 2007). In contrast, POT1b is implicated in chromosome end protection (Shakirov et al., 2005). Notably, AtPOT1a has a high binding specificity for the RNA subunit of telomerase (Cifuentes-Rojas et al., 2011), an unexpected mode of action for an OB-fold containing protein originally evolved to bind DNA. Unlike *A. thaliana*, but similar to the situation in *P. patens*, the *S. moellendorffii* genome encodes only a single POT1 protein. As expected from phylogenetic positions of their corresponding species, *S. moellendorffii* POT1 shares more amino acid similarity with *P. patens* POT1 (60%), than with *A. thaliana* POT1 proteins (46% to AtPOT1a and 47% to AtPOT1b; Shakirov et al., 2009b). Despite the overall higher amino acid conservation between *P. patens* and *S. moellendorffii* POT1 proteins, the loss of telomeric DNA binding capacity (Shakirov et al., 2009b) clearly suggests that the functional role of POT1 in *S. moellendorffii* telomere biology may in fact be more analogous to the situation in *A. thaliana* than in *P. patens*. Determining whether *P. patens* POT1 binds telomerase RNA must await the identification of this molecule in moss.

#### TRFL proteins

The second class of telomeric DNA binding proteins associates with ds telomeric DNA. This family of telomere repeat binding factors (TRF) shares a conserved Myb-related DNA-binding domain in the C-terminus and a central dimerization domain (Bilaud et al., 1996). Mammals and other vertebrates encode two ds telomere binding proteins, TRF1 and TRF2, with distinct functions in telomere homeostasis. TRF1 is thought to act primarily in telomere length control, while TRF2 is required for chromosome end protection through participation in T-loop formation (Broccoli et al., 1997; Griffith et al., 1999).

**FIGURE 2:**
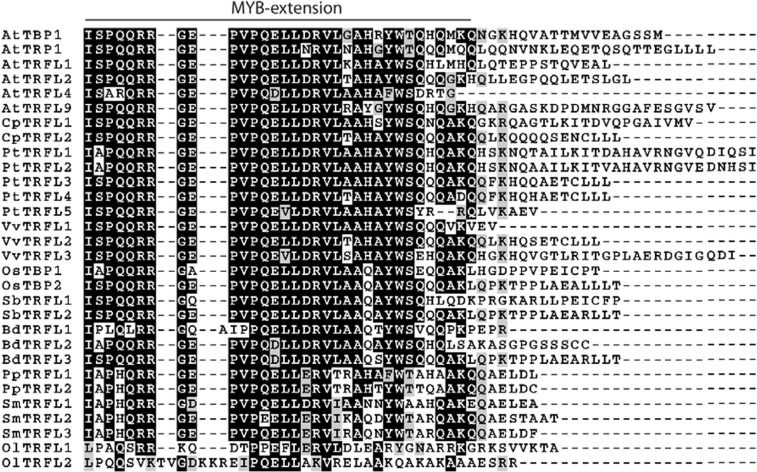
**Multiple alignment of C-terminal regions of plant group I TRFL proteins.** Position of conserved plant-specific MYB-extension motif is indicated. Abbreviations: At, *Arabidopsis thaliana*; Cp, *Carica papaya*; Pt, *Populus trichocarpa*; Vv, *Vitis vinifera*; Os, *Oryza sativa*; Sb, *Sorghum bicolor*; Bd, *Brachypodium distachyon; *Pp, *Physcomitrella patens*; Sm, *Selaginella moellendorffii*; Ol, *Ostreococcus lucimarinus*. Accession IDs: AtTBP1, NP_196886; AtTRP1, NP_200751; AtTRFL1, NP_190243; AtTRFL2, NP_172234; AtTRFL4, NP_190947; AtTRFL9, NP_187862; CpTRFL1, EU909205; CpTRFL2, EU909206; PtTRFL1, XM_002316243; PtTRFL2, XM_002311138; PtTRFL3, XP_002313432; PtTRFL4, XM_002299832; PtTRFL5, XM_002308126; VvTRFL1, CBI30542; VvTRFL2, CBI16113; VvTRFL3, CBI31661; OsTBP1, AF242298_1; OsTBP2, ABF95241; SbTRFL1, XP_002446657; SbTRFL2, XP_002468104; BdTRFL1, XP_003572947; BdTRFL2, XP_003565159; BdTRFL3, XP_003558235; PpTRFL1, XP_001771004; PpTRFL2, XP_001767955; SmTRFL2, XP_002979224.1; SmTRFL3, EFJ24726; SmTRFL1, XP_002984862.1; OlTRFL1, XP_001421395.1; OlTRFL2, XP_001416857.1. Protein alignment was generated using MEGA5 software (Tamura et al., 2011) and visualized in the BOXSHADE format.

Unlike vertebrates, plants possess two related families of TRF-like (TRFL) proteins, class I and class II (Chen et al., 2001; Hwang et al., 2001; Karamysheva et al., 2004). In *A. thaliana, *there are 12 TRFL proteins, 6 in class I and 6 in class II. Members of class II do not bind ds TTTAGGG repeats *in vitro*. In contrast, all six members of class I specifically bind ds telomeric DNA (Karamysheva et al., 2004). This interaction is dependent on the presence of a unique plant-specific Myb-extension motif, located at the extreme C-terminus of the TRFL protein (**Figure 2**). Overall, plants display remarkable variation in the number of class I *TRFL* genes. In dicot species, *TRFL* gene amplification appears to be a common theme, with three genes in grapes (*Vitis vinifera*), five genes in poplar (*Populus trichocarpa*), and six genes in *A. thaliana* (**Table 1**). In contrast, sequenced genomes of monocots and non-flowering plants, including *S. moellendorffii*, harbor 2 or 3 *TRFL* genes. While the precise role of individual TRFL proteins in plants remains unclear, amplification of *TRFL* gene family may provide a route for sub- and neo-functionalization with the potential for more dynamic control of telomere length or telomerase activity.

**Table 1:** Putative *TRFL* genes in selected plants with sequenced genomes.

Species	Plant lineage	Number of *TRFL* genes	Sequence IDs	References
*Vitis vinifera*	Dicot	3	CBI30542, CBI16113, CBI31661	
*Populus trichocarpa*	Dicot	5	XM_002316243, XM_002311138, XP_002313432,XM_002299832, XM_002308126	
*Carica papaya*	Dicot	2	EU909205, EU909206	Shakirov et al. (2008)
*Arabidopsis thaliana*	Dicot	6	NP_196886, NP_200751, NP_190243, NP_172234,NP_190947, NP_187862	Hwang et al. (2001), Chen et al. (2001) and Karamysheva et al. (2004)
*Oryza sativa*	Monocot	2	AF242298_1, ABF95241	Yu et al. (2000)
*Brachypodium distachyon*	Monocot	3	XP_003572947, XP_003565159, XP_003558235	
*Sorghum bicolor*	Monocot	2	XP_002446657, XP_002468104	
*Selaginella moellendorffii*	Spikemoss	3	XP_002979224.1, EFJ24726, XP_002984862.1	
*Physcomitrella patens*	Bryophyte	2	XP_001771004, XP_001767955	
*Ostreococcus lucimarinus*	Green alga	2	XP_001421395.1, XP_001416857.1	

**FIGURE 3:**
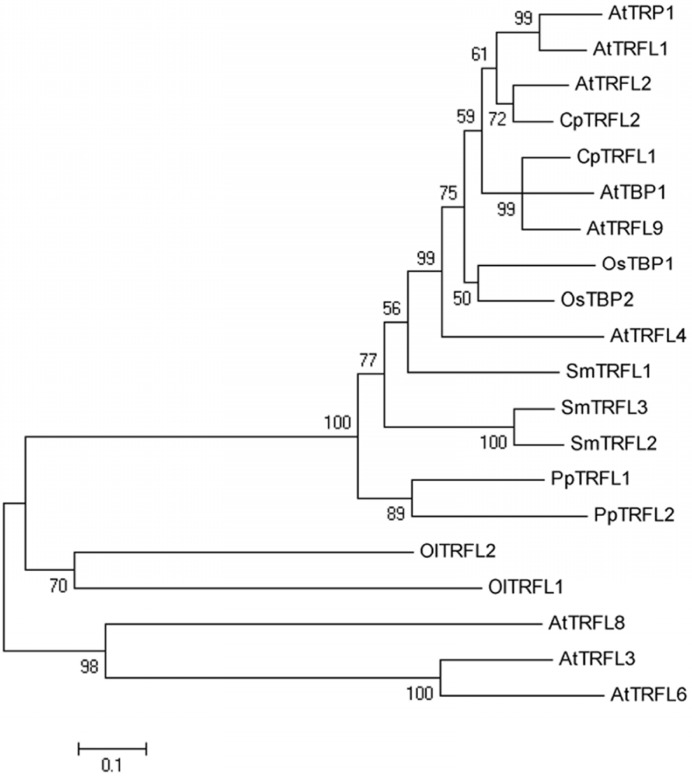
** Evolutionary relationships of plant group I TRFL proteins.** The evolutionary history of 17 currently available full-length TRFL proteins was inferred using the Neighbor-Joining method (Saitou and Nei, 1987). *A. thaliana* Group II TRFL proteins lacking the Group I-specific Myb-extension motif (AtTRFL3, AtTRFL6 and AtTRFL8) were used as outgroup (Karamysheva et al., 2004). The bootstrap consensus tree was inferred from 1000 replicates, and the percentage of replicate trees in which the associated taxa clustered together in the bootstrap test are shown next to the branches. The evolutionary distances were computed using the Poisson correction method (Zuckerkandl and Pauling, 1965). All positions containing gaps and missing data were eliminated. There were a total of 162 positions in the final dataset. Evolutionary analyses were conducted in MEGA5 (Tamura et al., 2011).

We also examined the evolutionary relationship of the available class I full-length plant TRFL proteins, using three *A. thaliana* class II proteins as the outgroup (**Figure 3**). As expected, class II proteins form a separate clade distinct from class I, consistent with the lack of the C-terminal Myb-extension motif. The evolutionary relationship of class I proteins correlates with the phylogenetic position of the corresponding plant species. Notably, the two TRFL proteins from green alga form a sister clade to all TRFL proteins from land plants, suggesting significant sequence divergence in this ancestral lineage.

#### CST components

A third group of evolutionarily conserved plant telomere proteins is a trimeric complex composed of CTC1, STN1, and TEN1, termed CST (Price et al., 2010). In the budding yeast *Saccharomyces cerevisiae*, a similar complex, composed of Cdc13, Stn1, and Ten1 proteins, was described over 20 years ago, but only recently has this complex come to light in multicellular eukaryotes (Wellinger, 2009). Individual CST components are highly divergent, with only 10–20% amino acid sequence identity between corresponding proteins from different eukaryotic lineages (Price et al., 2010). *A. thaliana* mutants deficient in *STN*1 and *CTC*1 are characterized by severe defects in telomere maintenance, massive end-to-end chromosome fusions, and elevated rates of telomere recombination (Song et al., 2008; Surovtseva et al., 2009). As in vertebrates (Casteel et al., 2009), the *A. thaliana* CTC1 subunit of CST physically interacts with the catalytic subunit of DNA polymerase α (Price et al., 2010), thus linking the CST complex to the telomere replication pathway.

To gain a better understanding of the evolution of CST complex in plants, we looked for the presence of genes encoding CST complex subunits in selected plant species with completely sequenced genomes. Single-copy *STN*1 and *TEN*1 genes were found in all organisms surveyed (**Table 2**). In addition, with the exception of the green alga *O. lucimarinus*, a single copy of *CTC*1 gene was also identified in all plant species analyzed, including *S. moellendorffii* (**Table 2**). The apparent absence of a clear *CTC*1 homolog in *O. lucimarinus* is intriguing. Strikingly, none of the CTC1 orthologs can be readily identified in several species of genera Chlamydomonas, Micromonas, and Chlorella, which belong to evolutionarily distinct lineages of green algae. These data indicate that either CTC1 sequence has diverged beyond recognition or that in green algae this protein has been functionally replaced by an unrelated polypeptide. This observation is in line with the observed sequence divergence of green algae TRFL proteins and suggests that many components of the telomere complex in green algae diverged substantially from their counterparts in land plants.

**Table 2:** Putative CST complex proteins from selected plants with sequenced genomes.

*Species*	STN1	TEN1	CTC1
*Vitis vinifera*	XP_003632321	CBI39529	CAN78397.1
*Populus trichocarpa*	EEE94597	EEF08821	XM_002302411
*Carica papaya*	JX198688	JX198687	JX198685
*Arabidopsis thaliana*	NP_563781.1	NP_176022.2	NP_001118960.1
*Oryza sativa*	EEE59110	EAZ38764.1	EEE69601.1
*Brachypodium distachyon*	XP_003557913	JX198686	JX198686
*Sorghum bicolor *	EER92221.1	EER95890.1	XP_002462303.1
*Selaginella moellendorffii*	EFJ12878	EFJ26866	XP_002963931.1
*Physcomitrella patens*	EDQ74366	XP_001782219.1	EDQ49834
*Ostreococcus lucimarinus*	ABO95476	ABO98342	ND*

*Ostreococcus lucimarinus CTC1 sequence could not be discerned.

Interestingly, it has been argued that the budding yeast *S. cerevisiae* replaced CTC1 with Cdc13 (Mitton-Fry et al., 2002), The two proteins share little sequence similarity, but possess structurally similar OB-fold DNA binding domains and interact with well-conserved protein binding partners (STN1 and TEN1). Our genome analysis indicates that single-copy genes encoding CST complex subunits are present in all land plants analyzed, from the earlier evolved non-seed plant lineages, represented by *P. patens* and *S. moellendorffii*, to the more developmentally advanced flowering plants. Thus, the important functions of CST complex in chromosome end protection and/or telomere replication are likely to be conserved throughout evolution of land plants.

## CONCLUSION AND OUTLOOK

Lycophytes occupy a unique phylogenetic position in the evolution of land plants, as they are ancient representatives of vascular plants and sister to Euphyllophites (which include flowering plants). Since most components of the telomere maintenance machinery have previously been analyzed only in Angiosperms, we examined the telomere repeat array and sequence homologs of telomere-related factors in *S. moellendorffii*. As in *A. thaliana* and *P. patents*, telomere tracts are short and consist of the canonical TTTAGGG repeat. Furthermore, similar to the situation in Angiosperms and other land plants, the *S. moellendorffii* genome harbors homologs of *POT*1, *TRFL, STN*1*, CTC*1, and *TEN*1 genes. Finally, our study revealed marked sequence divergence in telomere components of green algae relative to *S. moellendorffii*, arguing that future comparative studies among these organisms may provide important insight into the evolution of the telomere complex in plants.

## MATERIALS AND METHODS

### TELOMERE LENGTH ANALYSIS AND *Bal*31 DIGESTION

*Selaginella moellendorffii* DNA was extracted as described by Cocciolone and Cone (1993). To detect telomeric DNA repeats, genomic DNA was digested with *Tru*1I (Fermentas; recognition sequence TTAA) and subjected to Southern blotting with ^32^P-labeled (TTTAGGG)_4_ as a probe (Fitzgerald et al., 1999). Radioactive signals were scanned by a STORM PhosphorImager (Molecular Dynamics), and the data were analyzed by IMAGEQUANT software (Molecular Dynamics). For the *Bal*31 exonuclease assay, 100 µg of *S. moellendorffii* genomic DNA was incubated with 50 units of *Bal*31 (New England Biolabs) or with H_2_O (0 min time point) in 1× *Bal*31 reaction buffer at 30°C.Equal amounts of sample were removed at 15 or 30 min intervals for 90 min. Reactions were stopped by the addition of 20 mM EGTA and heating to 65°C for 15 min. DNA in each sample was precipitated with isopropanol and ammonium acetate, followed by *Tru*1I digestion. Digested DNA was separated on 0.8% agarose, blotted onto a nitrocellulose membrane and subjected to hybridization as described above.

### BLAST SEARCHES AND GENE PREDICTIONS

BLAST searches were performed at the Phytozome v8 portal^1^ using the tblastn option and amino acid sequence of *A. thaliana* telomere proteins as a query. *O. lucimarinus* BLAST was performed at the corresponding genome portal^2^ using the similar pproach. GenBank accession numbers of all proteins are given in **Tables 1 and 2**.

## Conflict of Interest Statement

The authors declare that the research was conducted in the absence of any commercial or financial relationships that could be construed as a potential conflict of interest.

## Footnotes

1 http://www.phytozome.net/

2 http://genome.jgi-psf.org/Ost9901_3/Ost9901_3.info.html

## ADDENDUM

The occurrence of *Arabidopsis*-type telomeres was previously shown by FISH in *Selaginella martensii* (J. Fuchs and I. Schubert “*Arabidopsis*-type telomere sequences on chromosome termini of *Selaginella martensii* Spring (Pteridophyta)” *Biol. Zent. Bl.* 115, 260–265, 1996).
